# Subcapsular Renal-Infected Hematoma After Retrograde Intrarenal Surgery: A Rare but Serious Complication

**DOI:** 10.1089/cren.2016.0007

**Published:** 2016-03-01

**Authors:** José A. Salvadó, Lucas Consigliere, Hector Gallegos, Francisco Rojas, Gastón Astroza

**Affiliations:** ^1^ Departamento de Urología, Hospital Clínico Pontificia Universidad Católica de Chile, Santiago, Chile.; ^2^ Servicio de Urología, Hospital Dr. Sótero del Río, Puente Alto, Chile.

## Abstract

We report a case of a 53-year-old woman affected by a left kidney stone and persistent positive urinary culture treated by retrograde intrarenal surgery. During postoperative day 1, she developed a sudden back pain associated with a decrease in hemoglobin. CT scan showed a subcapsular hematoma giving the impression of partial compression of kidney and upper urinary tract. For that reason, in the first instance, a Double-J ureteral stent was installed. Unfortunately, an open surgical drainage was necessary because a secondary infection of the hematoma was evident during the following days.

## Clinical History

A 53-year-old woman was admitted for an acute pyelonephritis. She was also suffering from obesity (body mass index: 30) and mild arterial hypertension. A CT scan revealed a renal abscess (4 cm max. diameter) associated with a stone impacted in the ureteropelvic junction in the left kidney (max. diameter: 17 mm; 500 HU). Initial treatment consisted of intravenous antibiotics and upper urinary tract decompression (Double-J stent). The patient was discharged home 10 days after admission and image control was done 2 months later, where complete abscess resolution was evident and the stone was still present in a similar situation. After three courses of adequate antibiotic therapy, blood inflammatory parameters were normalized but urinary culture remained positive (*Klebsiella pneumoniae*).

She returned to the hospital for a discussion of possible treatment alternatives. Given her obese condition and the low local effectiveness of our SWL equipment (stone-free rates 45%), the patient opted for retrograde intrarenal surgery (RIRS) as definitive treatment of stone. Due to the presence of the same bacteria in the culture, despite continued use of oral antibiotic, ciprofloxacin, the patient was admitted 4 days before surgery to receive intravenous antibiotic treatment (amikacin).

## Physical Examination

Before surgery, the patient had normal vital signs. On physical examination, she was normal, and she only complained about the catheter-associated discomfort.

## Intervention

**FIG. 1.**
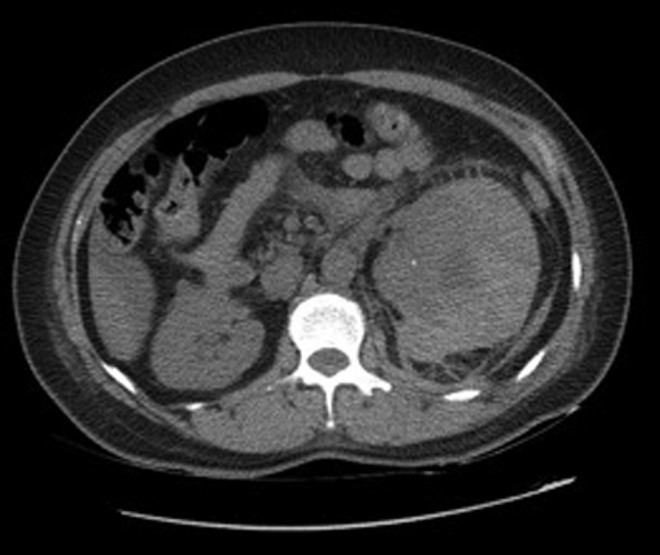
CT scan showing subcapsular hematoma with compression of the kidney.

**FIG. 2.**
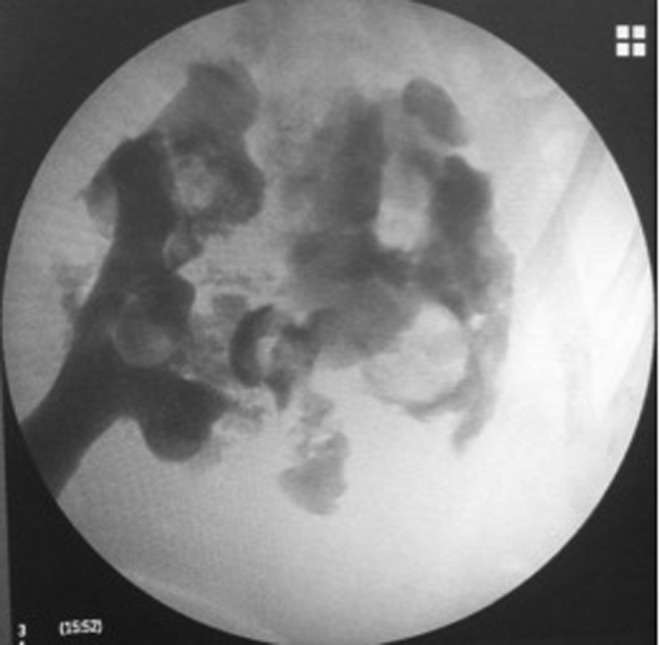
Retrograde pyelography with extravasation of contrast toward hematoma.

**FIG. 3.**
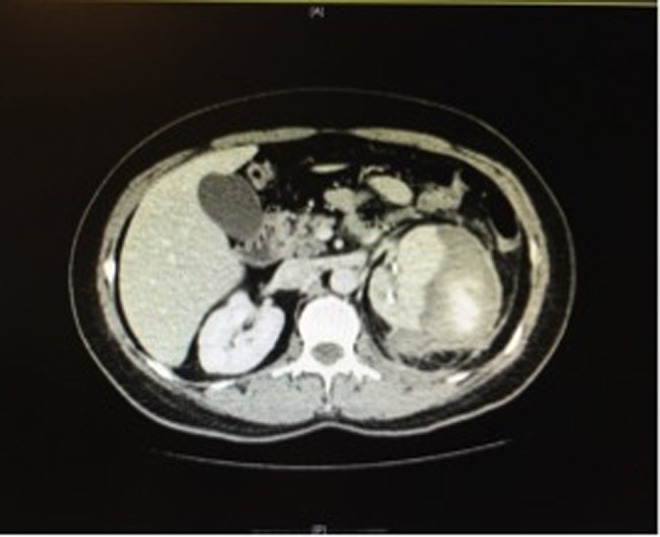
Infected hematoma with edema of peinephric fat tissue.

Under general anesthesia, RIRS was effectively performed. During the procedure, a 12/14F ureteral access sheath was used (Navigator^™^ HD; Boston Scientific) and the dusting technique for laser lithotripsy was performed. At the end of the procedure, a 6F open-end catheter and a Foley catheter were left in place. Irrigation method selected was the syringe with a flow rate of 40 mL/minute. Total operative time was 100 minutes. Both catheters were removed the morning of the next day; few hours later, the patient developed tachycardia, hypotension, and severe lumbar pain. Hemoglobin fell from 11 to 8 g/dL. Control CT scan showed a 4.1 cm subcapsular hematoma without active bleeding; considerable displacement of the kidney resulting in partial obstruction of the upper urinary tract and two nonobstructing stones fragments in the distal ureter (about 2–3 mm in diameter) were also seen (Fig. 1). Observation management was planned because the patient remained stable. At postoperative day 3, she developed fever with significant increase of C-reactive protein, creatinine, and leukocytosis of 16,000. We decided to change antibiotic therapy after infectious disease unit consultation (meropenem, based on sensibility) and reinstall a Double-J stent. Retrograde pyelogram showed contrast media extravasation in the upper pole calix with opacification of the hematoma, which impressed to be associated with urinary fluid collection; stone fragments in the distal ureter were no longer present (Fig. 2). At that point, our theory was that keeping low pressure in the collecting system with appropriate antibiotic therapy would be sufficient measures to deal with the infection. Her postoperative period was uneventful, with intermittent fever and inflammatory parameters unchanged. At postoperative day 20, an image control was done, showing hematoma without any differences concerning previous CT scans (Fig. 3). Since no clinical improvements were achieved with conservative measures and given the size of the hematoma, a flank open exploration was planned and done. Due to the possibility that a nephrectomy could be necessary, the first step was to identify and isolate renal vessels. Second, we proceeded to open Gerota fascia, outputting old hemorrhagic content, without evidence of active urinary leakage. A retroperitoneal drainage was left in place.

## Outcome

**FIG. 4.**
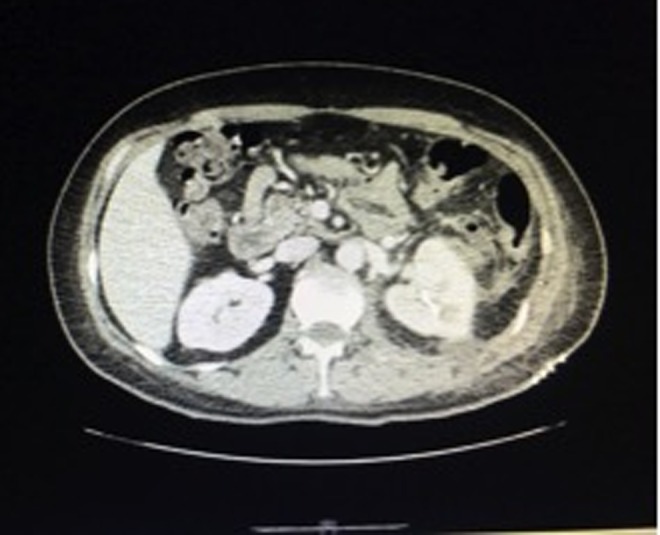
Complete resolution of the hematoma after open drainage.

The patient was discharged 20 days later, antibiotic treatment was indicated for 15 days at home. In outpatient control, a CT scan revealed total resolution of the hematoma (Fig. 4).

Post-RIRS, subcapsular hematoma is reported in less than 1% of patients,^1^ but its association with urinary leakage and infection has been reported in few studies. Bai and colleagues mentioned that post-RIRS, hematoma correlates with the use of high-pressure irrigation and the presence of severe hydronephrosis,^2^ which did not occur in our patient. Chiu and associates proposed that obese condition could increase difficulty in guidewire placement and ureteroscope advancement, thus increasing the chance of injury to the system.^1^ In this case, we can identify some risk factors for the complication described: prolonged time of surgery, the presence of residual stones in the ureter (which could increase the pressure within the urinary tract), and certainly the persistence of positive urine culture. To avoid postoperative sepsis, some points should be considered: in the first place, renal pelvic pressure should be kept as low as possible by using a large ureteral access sheath. Irrigation flow rate should also be low. Second, urinary tract infection should be resolved before definitive surgery. However, it is hard to achieve complete bacteria eradication in cases like the one we present. Optimal duration of antibiotic administration and the waiting time before carrying out RIRS remain to be established. Most patients who present with subcapsular hematoma could be treated conservatively, but when an infection occurs, the surgical approach should be more aggressive, including open drainage.

## Abbreviations Used

CT: computed tomography
HU: Hounsfield units
RIRS: retrograde intrarenal surgery
SWL: extracorporeal shockwave lithotripsy
